# Dimethyl itaconate protects against lipopolysaccharide-induced endometritis by inhibition of TLR4/NF-κB and activation of Nrf2/HO-1 signaling pathway in mice

**DOI:** 10.22038/ijbms.2020.44151.10346

**Published:** 2020-09

**Authors:** Mingyue Xu, Peng Jiang, Haowen Sun, Xin Yuan, Siyuan Gao, Jian Guo, Caijun Zhao, Xiaoyu Hu, Xueshibojie Liu, Yunhe Fu

**Affiliations:** 1 Department of Clinical Veterinary Medicine, College of Veterinary Medicine, Jilin University, Changchun, Jilin Province 130062, People, Republic of China; 2 Department of Otolaryngology, Head and Neck Surgery, the Second Affiliated Hospital of Jilin University, Changchun, China

**Keywords:** Dimethyl itaconate, Endometritis, LPS, NF-κB, Nrf2, TLR4

## Abstract

**Objective(s):**

Endometritis is the inflammation of the uterine lining that is associated with infertility. It affects milk production and reproductive performance and leads to huge economic losses in dairy cows. Dimethyl itaconate (DI), a promising chemical agent, has recently been proved to have multiple health-promoting effects. However, the effects of DI on endometritis remain to be unknown.

**Materials and Methods:**

In this study, we assessed the anti-inflammatory effects of DI on Lipopolysaccharide (LPS)-induced endometritis in mice. The endometritis was induced by LPS treatment for 24 hr, and DI was given 24 hr before induction of LPS.

**Results:**

As a result, DI administered mice significantly suffered less impairment of uterine tissue and less recruitment of inflammatory cells than LPS administered mice. In addition, DI markedly inhibited uterine myeloperoxidase (MPO) activity and pro-inflammatory cytokines of tumor necrosis factor alpha (TNF-α) and interleukin 6 (IL-6) induced by LPS. Moreover, LPS-induced toll-like receptor 4/ nuclear factor-kappa B (TLR4/NF-κB) activation was suppressed by DI. In addition, the expressions of nuclear factor erythroid 2-related factor 2 (Nrf2) and heme oxygenase (HO-1) were upregulated by DI.

**Conclusion:**

These findings suggest that DI has anti-inflammatory functions in the LPS-induced mice and may be a therapeutic agent against endometritis.

## Introduction

Bovine endometritis, an inflammation of the uterine caused by infection, is a disease associated with delayed uterine involution, which affects animal health (1). However, the cost of experimental study in cattle models is high and it is so difficult to manage. Therefore, mice are used as the model of bovine endometritis in recent years (2). Lipopolysaccharide (LPS), a major component of the outer membrane of gram-negative bacteria, is the pathogen-associated molecular pattern (PAMP) to the innate immune system. LPS can possess powerful biological functions and as an efficient stimulator in the immune system (3). It has been reported as an important virulence factor for bovine endometritis (2). Thus, an intrauterine infusion of LPS in a mouse model is used to study endometritis in laboratory.

Dimethyl itaconate (DI), the membrane permeable derivative of itaconate, is mainly used in paint, weak acid ion exchange resin, lubricating oil additive, binder, plasticizer, powdered plastic and sealant. It can selectively restrain a range of cytokines, such as interleukin 6 (IL-6) and IL-12. It has been confirmed that DI-treatment led to the reduction of a series of pro-inflammatory transcripts and inhibited the production of IL-1**β** and IL-18 induced under prototypical NLRP3-activating conditions (4, 5). There is growing evidence suggesting that DI and itaconate inhibit the activity of bacteria expressing isocitrate lyase and have an important role in tumor progression (6, 7). It has also been proved that DI activates Nrf2 and increases the level of nuclear factor erythroid 2-related factor 2 (Nrf2) protein (8). Furthermore, several studies indicated that DI was associated with the inflammatory responses in macrophages and other immune cells (9). However, none of these studies have investigated the anti-inflammatory effects of DI on LPS-induced endometritis in mice. Thus, in this study, we tested the protective effects and anti-inflammatory mechanisms of DI on endometritis induced by LPS.

## Materials and Methods

***Chemicals and reagents***

Lipopolysaccharide (LPS, *Escherichia coli* 055:B5) and DI (purity ≥ 99%) were purchased from Sigma-Aldrich (St. Louis, MO, USA). Primary antibodies for β-actin, toll-like receptor 4 (TLR4), Nrf2, heme oxygenase (HO-1) and phosphorylated and non-phosphorylated forms of nuclear factor-kappa B (NF-κB) were purchased from Cell Signaling Technology Inc. (Beverly, MA, USA). All other chemicals were of reagent grade.

***Mouse model of LPS-induced endometritis***

Sixty female BALB/c mice (6-8 weeks old) were obtained from the Center of Experimental Animals of Baiqiuen Medical College of Jilin University (Jilin, China). The animals were raised for 1 week with free access to water and food to adapt to the feeding environment. All procedures were conducted according to the US NIH Guidelines for the Care and Use of Laboratory Animals. In previous study, scholars had explored an optimal method to build up the endometritis model by LPS (10-12). They infused each uterus with 20 μl of 2.5 mg/ml LPS or 50 μl of 1 mg/ml, which was 50 μg LPS in total. As a reference, we built up the mouse endometritis models at the same conditions. The mouse endometritis model was established by injection of LPS (2.5 μg/μl) dissolved in sterile phosphate-buffered saline (PBS) into the uterus (13). Each mouse uterus was injected 20 μL of LPS to induced endometritis. The 60 female BALB/c mice were randomly divided into six groups as follows: the blank control group, the DI administration group, the LPS group, and DI (5, 10 or 20 mg/body weight) + LPS groups. DI was solubilized by PBS to obtain final concentrations of 5, 10 or 20 mg/body weight. DI was given IP 24 hr before LPS treatment in previous research (4). At 24 hr after LPS treatment, the mice were anesthetized with pentobarbital sodium and sacrificed by CO_2 _inhalation. Then, uterine tissues were collected and kept at -80 °C until analysis. The experiments were carried out in accordance with the guidelines of the National Institutes of Health for the care and use of Laboratory animals.

***Histopathological assessment***

Twenty-four hour after infusion of LPS, the uterus tissues were isolated and fixed in fresh 4% formaldehyde solution for 24 hr, then embedded in paraffin, sliced, later stained with hematoxylin and eosin (H&E). Finally, the histological changes in the uterus tissues were observed with a light microscope (Olympus, Japan).

***MPO activity analysis***

Uterus tissues were collected 24 hr after the LPS challenge, then weighed and homogenized with reaction buffer (w/v 1/9). Myeloperoxidase (MPO) activity was measured with the commercial kits in accordance with the manufacturer’s instructions (Nanjing Jiancheng Bioengineering Institute, China).

***Analysis of cytokines***

The uterine tissues were weighed and homogenized with PBS (w/v 1:9) on the ice, and then centrifuged at 3000 g for 10 min at 4 **°**C. The tissue supernatants were harvested to detect the expression of IL-1β and tumor necrosis factor alpha (TNF-α) using ELISA kits according to the manufacturer’s instructions (BioLegend, CA, USA).The absorbance value was measured at 450 nm with a microplate reader.

***Western blot analysis***

The uterus tissues were weighed and homogenized with tissue lysis buffer containing the cocktail protease inhibitor (w/v 1:9) on ice. The mixture was centrifuged at 12000 g, 4 °C for 10 min. The protein concentrations were determined using a BCA protein assay kit. (Thermo Scientific, MA, USA) Protein extracts were separated by SDS-PAGE and the proteins were then electrotransferred onto polyvinylidene fluoride (PVDF) membranes. The membranes were incubated with 5% skim milk in Tris Buffered Saline Buffer with Tween 20 (TBST) for 3 hr. After incubation at 4 **°**C with the primary antibodies (1:1000–1:5000 dilutions) overnight, and then washing 3 times for 15 min each time with TBST, a secondary horseradish peroxidase (HRP)-conjugated anti-rabbit IgG antibody (1:20,000) was applied at room temperature for 2 hr. After washing three times with TBST, the protein levels were observed using the Bio-rad Imaging System (Bio-rad Biosciences, USA). The β-actin was used as the internal control for total protein.

***Statistical analysis***

All data were expressed as the mean±SEM. The images were produced using GraphPad Prism software and the mice were randomly assigned to groups. All statistical analyses were performed using the unpaired Student’s *t*-test and one-way analysis of variance (ANOVA) with Tukey *post hoc* test. The results were considered statistically significant at *P*<0.05, *P*<0.01 or *P*<0.001.

## Results

***Effects of DI on LPS-induced uterine tissue histopathological changes***

**Figure 1:**
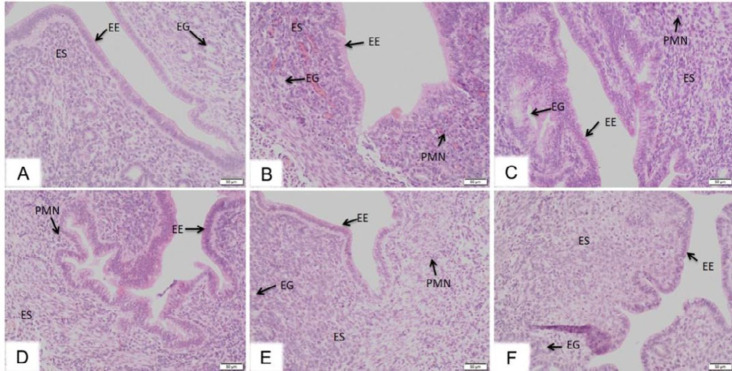
Effects of DI on LPS -induced uterus histopathologic changes. Twenty-four hour after the perfusion of LPS, the histopathological features of the uterine tissues were observed by H&E staining. Pathological changes in the uterine structures of a mouse in the control group (a), the LPS model group (b), the LPS+ DI group (5 mg/body weight) (c), the LPS+ DI group (10 mg/body weight) (d), the LPS+ DI group (20 mg/body weight) (e), and DI group (20 mg/body weight) (f) (magnification × 200). Endometrial epithelium (EE), endometrial gland (EG), endometrial stroma (ES) and polymorphonuclear (PMN). DI: dimethyl itaconate, LPS: lipopolysaccharide

Histopathological changes in uterine tissues from each group were examined by H&E staining. As shown in Figure 1a, there was no pathological change in the uterine tissue in the control group. As for LPS group, inflammation cells infiltrated into the endometrial epithelium, and inflammatory hyperemia, villi atrophy and epithelial cell destruction in endometrial epithelial tissues of mice was markedly increased compared to those of mice in the control group (Figure 1b). However, treatment of DI dose-dependently alleviated these changes induced by LPS (Figure 1c–1e). In addition, compared to the damage in the LPS group, histological analysis displayed normal morphology and only a small amount of leukocyte infiltration was observed in the DI group (Figure 1f).

***Effects of DI on MPO activity during LPS-induced endometritis***

**Figure 2:**
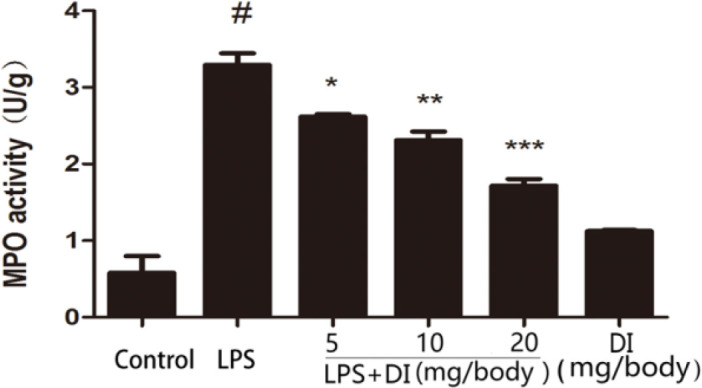
Effects of DI on the MPO activity. Mice were administered DI (5, 10, and 20 mg/body weight, IP) 24 hr before LPS induction. The MPO activity was determined 24 hr after LPS induction. The values presented are mean±SEM of three parallel measurements. *P*-value<0.001:#vs. the control group. *P*-value<0.001:***, *P*-value<0.01:**and *P*-value< 0.05:* vs. the LPS group. DI: dimethyl itaconate, LPS: lipopolysaccharide, MPO: myeloperoxidase

MPO activity serves as a biomarker of neutrophils infiltration at the site of inflammation. The results showed that LPS challenge caused a great increase in MPO activity compared to the control group. However, treatments of DI dose-dependently inhibited LPS-induced production of MPO activity. In addition, DI group had no significant differences compared to the control group (Figure 2).

***Effects of DI on pro-inflammatory cytokines in uterine tissues of LPS-induced endometritis model mice***

**Figure 3:**
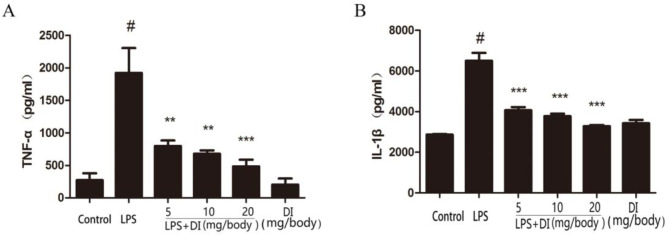
Effects of DI on the levels of TNF-α (A) and IL-1β (B) in LPS-induced endometritis. Mice were administered DI (5, 10, and 20 mg/body weight, IP) 24 hr before LPS induction. The levels of inflammatory cytokine expression in tissue homogenates were calculated using ELISA. The values presented are mean±SEM of three parallel measurements. *P*-value<0.001:# vs. the control group. *P*-value<0.001:***and *P*-value<0.01:**vs. the LPS group. DI: dimethyl itaconate, LPS: lipopolysaccharide, TNF-α: tumor necrosis factor alpha, IL-6: interleukin 6

As shown in Figure 3, the levels of TNF-α and IL-1β in the LPS group were extremely higher than the control group. However, the levels of pro-inflammatory mediators were dose-dependently inhibited by DI. In addition, there were no significant differences between the control and DI group (Figure 3).

***Effects of DI on the activation of NF-κB signaling pathway in LPS-induced mice endometritis***

**Figure 4:**
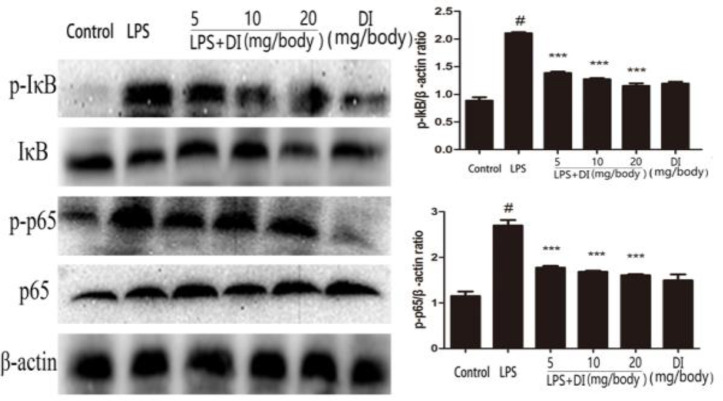
Effects of DI on LPS-induced NF-κB activation. Mice were administered DI (5, 10, and 20 mg/body weight, IP) 24 hr before LPS induction. The expression of NF-κB p65, NF-κB pp65, IκB, and p-IκB were detected by western blot. β-actin was used as a control. The values presented are mean ± SEM of three parallel measurements. *P*-value< 0.001:#vs. the control group and *P*-value< 0.001:***vs. the LPS group. DI: dimethyl itaconate, LPS: lipopolysaccharide, NF-κB: nuclear factor-kappa B

In the present study, the levels of NF-κB signaling pathway proteins were detected by Western blot. The phosphorylated inhibitor of nuclear factor kappa B (IκBα) and NF-κB p65 proteins were dramatically increased in the LPS group compared to the control group. On the contrary, DI (5, 10 or 20 mg/body weight) treatment significantly inhibited the LPS-induced expression of p-IκB and p-p65 in uterine tissues (Figure 4).

***Effects of DI on the expression of TLR4 in LPS-induced mice endometritis***

**Figure 5:**
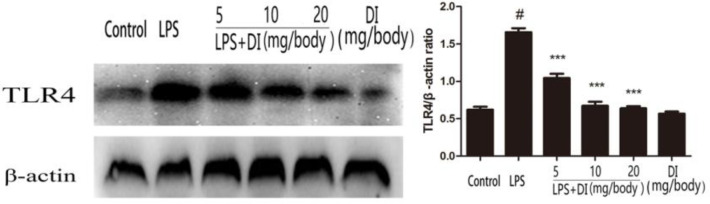
Effects of DI on TLR4 expression. Mice were administered DI (5, 10, and 20 mg/body weight, IP) 24 hr before LPS induction. The expression of TLR4 was detected by western blot. β-actin was used as a control. The values presented are mean±SEM of three parallel measurements. *P*-value< 0.001:#vs. the control group and *P*-value< 0.001:***vs. the LPS group. DI: dimethyl itaconate, LPS: lipopolysaccharide, TLR4: toll-like receptor 4

The results showed that the expression of TLR4 in the LPS group was significantly higher than the control group. However, the DI also produced significant inhibition of the TLR4 levels in uterine tissues that were induced by LPS. The results are shown in Figure 5.

***Effects of DI on the expression of Nrf2 and HO-1 in LPS-induced mice endometritis***

**Figure 6:**
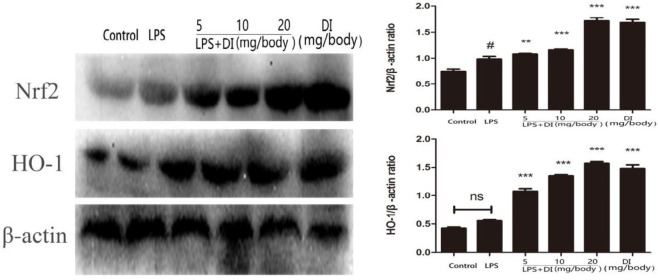
Effects of DI on the expression of Nrf2 and HO-1. Mice were administered DI (5, 10, and 20 mg/body weight, IP) 24 hr before LPS induction. The expression of Nrf2 and HO-1 were detected by western blot. β-actin was used as a control. The values presented are mean ± SEM of three parallel measurements. *P*-value< 0.05:#vs. the control group. *P*-value< 0.001:*** and *P*-value<0.01:**vs. the control group. DI: dimethyl itaconate, LPS: lipopolysaccharide, Nrf2: nuclear factor erythroid 2-related factor 2, HO-1: heme oxygenase

We measured the effects of DI on Nrf2 signaling pathway and the results showed that DI significantly activated the Nrf2 defense pathway, where it boosted Nrf2 and HO-1 levels compared to control group. In addition, we found that the expression of Nrf2 was upregulated by LPS (Figure 6).

## Discussion

Bovine endometritis is mainly due to vaginal or cervical infection with certain pathogenic microorganisms and varying degrees of trauma infection of a reproductive tract disease. It is a common disease in the postpartum period in cattle (14, 15). Postpartum endometrial inflammation affects milk production and reproductive performance in dairy cows and causes serious economic loss in the dairy industry (16, 17). DI, an important derivative of itaconic acid ester, has recently emerged as a regulator of macrophage function. In previous studies, it has been reported that DI has anti-inflammatory action and plays a vital role in supporting immune function (18). Although some studies have been reported on the anti-inflammatory effects of DI (19-21), its effect and mechanism in protection against endometritis are not entirely clear. Moreover, the way to build up the mouse endometritis models has been performed after many experiments. Thus, we used mice as the model of bovine endometritis to investigate the protective effects of DI on this disease.

In the present study, histological analysis by H&E staining showed that the LPS-induced inﬂammatory cell infiltration was reduced and the structure of the uterus was relatively intact after pretreatment with DI. It is displayed that DI significantly attenuated the damage of uterine tissues. MPO is a hemoprotein secreted during activation of neutrophils, and it is very vital in the defense of body. It can also reflect the level of neutrophils at the site of inflammation (22). The result showed that pretreatment with DI could reduce the levels of MPO significantly. Pro-inflammatory cytokines play a crucial role in the development of inflammation (23). In our study, we detected the levels of TNF-α and IL-1β by ELISA. The results showed that DI exerted its anti-inflammatory effects by inhibiting the production of TNF-α and IL-1β induced by LPS.

NF-κB signaling pathway plays an important role in inflammation. It has been reported to promote the production of pro-inflammatory cytokines, such as IL-1β, TNF-α, and IL-6 (24, 25). IκBα is a known inhibitor of NF-κB. However, upon NF-κB pathway activation, NF-κB binds to the IκBα DNA sequences and initiates gene transcription and expression. IκBα is phosphorylated and then degraded (26)Once NF-κB activated, the NF-κBp65 subunit translocates from the cytoplasm to the nucleus (27). During the LPS-induced inflammatory process, TLR4 plays a vital role as the receptor of LPS (28). Western blot results indicated that DI exerted its protective effects on LPS-stimulated endometritis by suppressing TLR4/NF-κB signaling pathway.

Nrf2 is a key mediator of endogenous inducible defense systems and stimulates the transcription of several antioxidant enzymes. It regulates the gene expression of a wide variety of cytoprotective phase II detoxifying proteins, such as HO-1 and nicotinamide adenine dinucleotide phosphate: quinine oxidoreductase-1 (NQO1) (29). The Nrf2 pathway is associated with the cellular response to various diseases such as arteriosclerosis thrombosis, endometritis and neuroinflammation (30-32). Recently, many findings have shown that activation of Nrf2 could alleviate LPS-induced inflammatory response (33), and Nrf2/HO-1 pathway was linked to the decreased levels of NF-κB expression (34). Others also suggested that the activation of Nrf2 and induction of HO-1 have both anti-inflammatory and antioxidant effects (35). In our study, we confirmed that DI reduced the degree of injury in LPS-induced endometritis by activation of Nrf2 pathway.

## Conclusion

In summary, the results of the present study indicated that DI exerted anti-inflammatory effects in LPS-induced mice endometritis. The protective mechanisms of DI were associated with inhibition of the production of pro-inflammatory cytokines by suppression of TLR4/NF-κB and activation of Nrf2 pathways. Our study adds to the accumulating evidence that suggests DI may be used as a potential medicine for curing endometritis in dairy cows.
